# Effect of 30-day Ramadan fasting on autophagy pathway and metabolic health outcome in healthy individuals

**DOI:** 10.22099/mbrc.2024.50105.1978

**Published:** 2025

**Authors:** Sanaz Dastghaib, Morvarid Siri, Nasim Rahmani-Kukia, Seyed Taghi Heydari, Mehdi Pasalar, Mozhdeh Zamani, Pooneh Mokaram6, Kamran Bagheri-Lankarani

**Affiliations:** 1 Endocrinology and Metabolism Research Center, Shiraz University of Medical Sciences, Shiraz, Iran; 2 Autophagy Research Center, Shiraz University of Medical Sciences, Shiraz, Iran; 3 Department of Biochemistry, School of Medicine, Shiraz University of Medical Sciences, Shiraz, Iran; 4 Health Policy Research Center, Institute of Health, Shiraz University of Medical Sciences, Shiraz, Iran; 5 Research Center for Traditional Medicine and History of Medicine, Shiraz University of Medical Sciences, Shiraz, Iran; 6 Autophagy Research Center, Department of Biochemistry, School of Medicine, Shiraz University of Medical Sciences, Shiraz, Iran

**Keywords:** Ramadan, Intermittent fasting, Health, Autophagy, Inflammation

## Abstract

During Ramadan, Muslims fast to spiritually prepare their bodies and spirits. The autophagy pathway restores cellular homeostasis and is being studied as a therapy for a variety of disorders. According to previous studies, fasting or calorie restriction has a role in the up-regulation of autophagy especially through the initiation step. The effects of Ramadan fasting on the autophagy pathways and metabolic health outcome in healthy adults were investigated in this study. In this controlled cohort study, 50 healthy subjects (20-78 years old) 24-fasting and 26 non-fasting were included. At the end of Ramadan, a blood was drawn to assess biochemical, hematological, and inflammatory variables. Serum IL-6 and *hs*-CRP levels were measured. The serum proteins (Beclin-1 and LC3β) and mRNAs gene expressions’ (*Beclin-1*, *p62*, and *LC3β*) of the autophagy marker were evaluated by ELISA and real-time PCR, respectively. During Ramadan, there were no significant differences for biochemical parameters (except for BUN-level), inflammatory markers (IL-6 and *hs*-CRP), and hematological indices during Ramadan. *Beclin-1* gene expression was significantly upregulated in the fasting-group as the main marker of initiation of autophagy; yet, the *LC3β* and the *p62* levels were decreased in the fasting-group in peripheral blood mononuclear cells. However, fasting women alone displayed a significantly high serum Beclin-1 level. Ramadan fasting does not have any adverse effects on biochemical, hematological, and inflammatory parameters. According to our results, people observing Ramadan may benefit from the autophagy pathway to compensate reduction in energy and vital metabolites in the face of food restriction.

## INTRODUCTION

Many religions observe fasting, which involves abstaining from food and drink at certain periods [1]. Muslims worldwide fast from dawn to sunset during Ramadan [2]. Time-restricted feeding/eating (TRF), Alternate-day fasting (ADF), intermittent energy restriction (IRE), and Ramadan IF are all variants of intermittent fasting. All entail extended fasting periods longer than an overnight fast and restricted eating windows with or without calorie restriction [3]. Ramadan fasting affects biochemical indicators in hypertension, metabolic syndrome, hypercholesterolemia, obesity, type 2 diabetes, and CKD [4]. 

Ramadan fasting affected glucose homeostasis, body weight, composition, fat mass, blood lipids, immunomodulation, and inflammation. Results from rodent trials indicate improved insulin sensitivity, glucose tolerance, and β-cell mass. The systematic review, meta-analysis, and meta-regression examined how Ramadan fasting affects glucose homeostasis, body weight, body composition, fat mass, serum lipids, immunomodulatory responses, and inflammatory and oxidative stress. In healthy people, Ramadan fasting showed little influence on glucometabolic indicators. The study also found that sex, age, fasting duration, and nationality affected glucometabolism [5, 6]. The randomized trial of 44 college students examined how Ramadan fasting affected body composition and metabolic syndrome in seemingly healthy men. No substantial changes in body composition were identified before, during, or after Ramadan fasting. One positive finding was a decrease in LDL values during Ramadan. Ramadan fasting may improve cardiovascular health by decreasing LDL [7].

Ramadan fasting lowers T2DM patients' blood glucose, lipid profile, blood pressure, HbA1c, BMI, and sleeping disorders [8]. Fasting and calorie restriction are linked to longer life and stress tolerance [9]. Urooj, Asna et al. found that Ramadan fasting with dietary awareness before fasting may improve spiritual and overall well-being. They found that weight loss improved HDL and liver function tests. Cytokines and sleep patterns may improve liver function tests[10]. Suzuki, K. found that fasting keeps us young and healthy. He claimed that during a fast, cells recycle unwanted material and generate new by 'autophagy' [11]. 

Recent studies show that fasting regulates glycolysis, Krebs cycle, apoptosis, and autophagy metabolites [12]. Autophagy, which involves lysosomal degradation, helps cells digest damaged organs, unfolded proteins, and invading microbes [13, 14]. Evolutionarily conserved proteins form autophagy's basic mechanism. This method supplies critical metabolites to cells during fasting, promoting health and longevity. The double-membrane autophagosomes produced by autophagy-related genes (ATGs) destroy cellular components in the lysosome [15]. This mechanism is essential for organelle turnover, nutrition sensing, protein and organelle breakdown, and cell survival. The double-edged blade route autophagy has been studied for a decade [16, 17]. Numerous studies have shown that the autophagy response to stress and starvation has two phases: first, autophagy is rapidly induced within minutes or seconds of stress exposure; second, specific pathways are activated to the extent that specific genes involving other pathways in the cell, such as UPR (unfolded protein response) and apoptosis, are transcribed [18]. Stressful stimuli such hypoxia, nutritional shortage, DNA failure, and cytotoxic agents induce autophagy, which affects cell death, aging, immunity, and metabolism [19]. Beclin-1 is essential for autophagy initiation and increases with stress. The BECN gene expresses Beclin-1, the first autophagy-related protein [20], which is necessary for embryonic development [21]. Additionally, LC3β and P62 are important indicators of autophagosome formation and autophagy flux in mammalian cells. Nutritional restriction may regulate autophagy and boost anti-cancer therapy while protecting normal cells [22]. 

Time-restricted feeding modulates central and peripheral circadian rhythms, so glycogen and lipid components can be used as fuel by increasing energy metabolism and autophagy genes. IF for 12-14 hours per day for 30 days increased serum proteomic anti-cancer levels, glucose, fat metabolism, insulin signaling pathway, biological clock, and DNA remodeling, treating obesity, T2DM, metabolic syndrome, inflammation, Alzheimer's, and several psychiatric conditions [23]. 

It has been accepted that inflammation and autophagy are linked. Activated autophagy degrades inflammatory mediators and clears damaged organelles. TNF-α, TGF-β, IL-1, IL-2, IL-6, and IFN-γ have been shown to trigger cell receptor-mediated autophagy. Ramadan fasting has been shown to alleviate inflammation and boost the immune system by regulating IL1β, IL-6, TNFα, and total monocytes, leukocytes, granulocytes, and lymphocytes in healthy individuals [24]. It has been reported that Ramadan fasting reduces circulating inflammatory parameters and improves the immune system by modulating IL1*β*, IL-6, and TNFα (tumor necrosis factor *α*), as well as total monocytes, leukocytes, granulocytes, and lymphocytes in healthy subjects [25]. Some systematic evaluations show that Ramadan fasting lowers body weight, fasting blood glucose, and cholesterol [26, 27], whereas others show the opposite. Ramadan fasting can cause mood swings, poor memory, poor work performance, interrupted sleep and metabolism, insulin sensitivity issues, and adipokine release [28, 29]. 

The authors are unaware of any studies on the effects of one-month Ramadan fasting on autophagy and inflammatory indicators in healthy people. Thus, our controlled cohort study examined how Ramadan fasting may influence these pathways to fill the knowledge vacuum. We want to study Ramadan's effects on biochemical and hematological parameters and the role of autophagy factors in serum and PBMC to assess cell function/metabolism, immune system, and inflammation in healthy fasting people.

## MATERIALS AND METHODS

### Study design and sampling:

**Figure 1:**
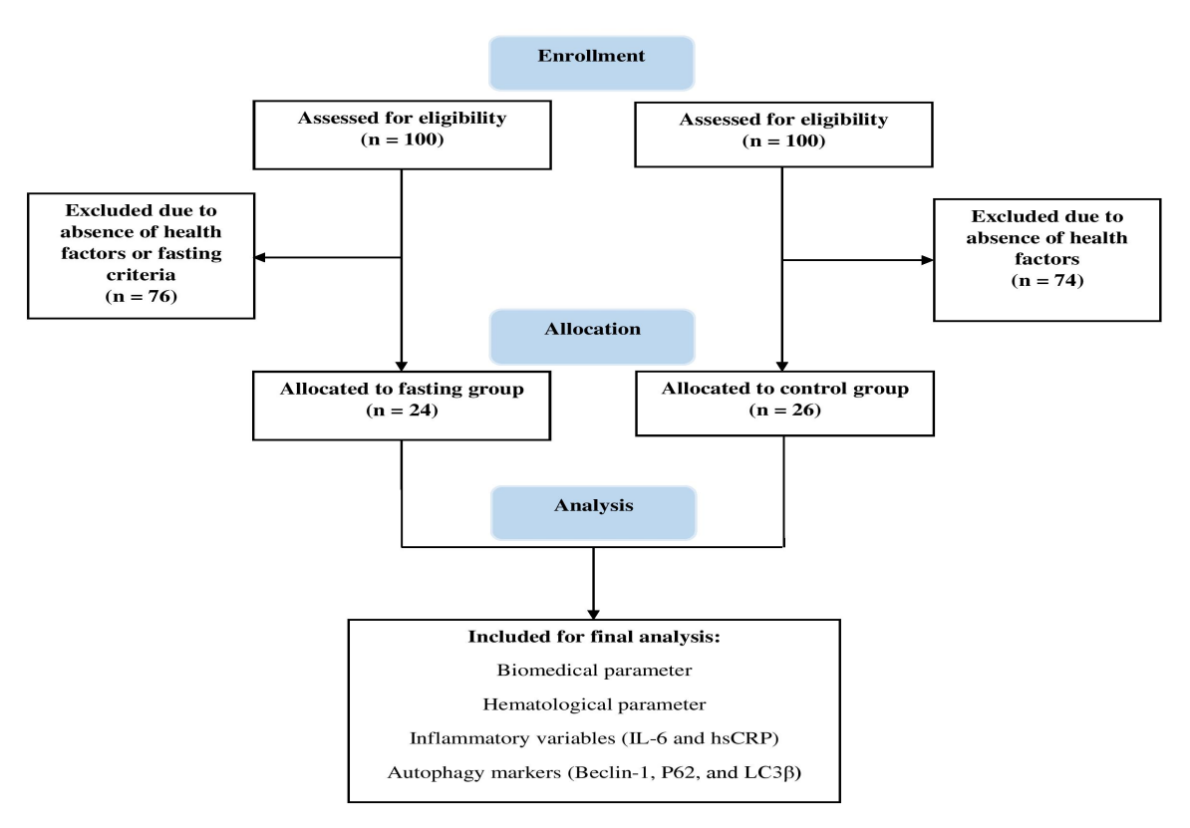
CONSORT flowchart of the participants

This controlled cohort study was conducted at the School of Medicine Shiraz University of Medical Sciences (SUMS) a day after Ramadan. This study conformed to the ethical standards of the Declaration of Helsinki. For this purpose, men and women volunteers, aged 20-78 years old were selected and studied at the SUMS on 16 May 2021 (a day after the holy month of Ramadan) (Fig.1).

Fasting group selection: The participants had to meet two specific criteria: they had to entirely fast during Ramadan and be at least 20 years old. Smokers, kidney diseases, T2DM, or malignant tumors were excluded from the study. Initially, there were 100 volunteers eligible for the project. However, during the primary screening, 76 of these individuals were excluded because they did not meet the specific health and fasting criteria. From the remaining pool, 24 individuals were included in the study, consisting of 14 males and 10 females.

Non-fasting group selection: we chose 26 out of the initial 100 volunteers. These 26 individuals were carefully selected to meet our inclusion criteria. The resulting of non-fasting group comprised 11 males and 15 females. We used to post online through popular social media platforms in Iran (Instagram, WhatsApp, and Telegram). First, all participants were requested to read and sign an informed consent form and then the research process was thoroughly explained to them. Their mean age was 42.0 years (SD, 10.7), a range of 20-78 years. Questionnaires were evaluated to ascertain the subjects’ physical activity level. A blood sample (20 ml) was obtained from each participant one day after Ramadan after at least 8 hours of fasting state in the morning (8 am). 20 ml of samples were divided into 3 separate parts for gene expression analysis via isolation of peripheral blood mononuclear (PBMC) cells (10 cc), for evaluating biochemical factors in serum (8 cc), and for hematological analysis from whole blood samples (2 cc). 

### Human PBMC isolation:

To isolate PBMC, which contain several types of cells such as lymphocytes, monocytes, or macrophages, we used a heparinized blood sample. The method for PBMC isolation on the Ficoll gradient was first described in 1968 [30, 31]. Briefly, whole blood was diluted with an equal volume of PBS. PBMCs were isolated using Ficoll (Lymphodex, Inno-Train, Germany) density-gradient separation. Then it was centrifuged at 700×*g*, at 4 °C for 20 min. PBMCs were collected from the buffy-coat layer and washed (500×*g*, 5 min, 4 °C) two times with PBS and kept at -80°C for further experiments. 

### Evaluation of autophagy markers:

 The total RNA was isolated from PBMC samples by the RNX-Plus RNA extraction kit (cinnagen, IRAN), following the producer’s instruction. Then, the RNA quantity and quality were assessed by electrophoresis and a nano-drop spectrophotometer (Thermo Scientific, USA) (260/280 nm ratio). cDNA was made by the cDNA Synthesis Kit (Thermo Fisher Scientific, USA) and amplified in the presence of specific primers. The *LC3* β, *P62*, *Beclin-1,* and *GAPDH* primers were obtained from Metabion Company (Germany) [14, 32]. The sequence of the primers is presented in the supplementary file (Table S1). Quantitative RT-PCR was performed in an ABI real-time PCR 7500 system (Applied Biosystems, USA). *GAPDH* (Glyceraldehyde-3-Phosphate Dehydrogenase) was considered as a reference control. The relative quantity of the target genes was calculated, using the delta-delta Ct (2^– ΔΔCT^) method. 

### Evaluation of serum biochemical, inflammatory, and autophagy parameters:

Divided 4 cc blood samples of all subjects were centrifuged at 3500 rpm for 10 minutes, then the serum was collected as soon as possible and stored at -70° C till further analysis. We measured lipids profile; triglyceride, cholesterol, high-density lipoprotein, and low-density lipoprotein (TG, Chol, HDL, and LDL), fasting blood sugar (FBS), blood urea nitrogen (BUN), Creatinine (Cr.), liver function test (LFT) including; alanine aminotransferase, aspartate aminotransferase, and alkaline phosphatase  (ALT, AST, and ALP), albumin, globulin, total protein, bilirubin (direct and indirect), calcium (Ca), phosphate (P), and hs-CRP using colorimetric assays and diagnostic colorimetric kits (BioSystem, Spain) by Mindray Bs 480 (Shenzhen Mindray Bio-Medical Electronics Co., Ltd., China) clinical auto-analyzer.

To evaluate the amount of interleukin 6 (IL6) (pg/ml), we used a solid phase, enzyme-labeled chemiluminescent immunometric assay (IMMULITE 2000; Siemens Healthcare, Netherlands). Serum Beclin-1(pg/ml) and LC3β II (pg/ml) were checked using the ELISA method by sandwich technology according to the manufacturer’s instructions (Sunlong company, China). For Beclin-1 with 14 pg/ml sensitivity, intra-and inter coefficient of variation (CVs) were <10% and <12%, and for LC3β II with 0.8 pg/ml sensitivity, intra-and inter CVs were <10% and <12%, respectively.

### Evaluation of hematological parameters:

To analyze complete blood count (CBC), ethylene-diamine-tetra-acetic acid (EDTA) - anticoagulated whole blood was taken using a standard method by Sysmex KX-21N automated hematology analyzer. Red blood cell (RBC), white blood cells (WBC), platelet (PLT) count, and hemoglobin (Hb) amount were measured. Another whole blood sample was taken to analyze glycosylated hemoglobin (HbA1c) by a High-Performance Liquid Chromatography (HPLC) module consisting of an Agilent 1100 Series (Agilent Technologies, Yokogawa Analytical System Inc., Tokyo, Japan) equipped with a UV detector for chromatographic analysis.

### Statistical analysis:

The primary endpoints of change in hsCRP as a serum inflammatory marker were utilized to calculate sample size. According to a pilot study of 15 participants, including fasting and non-fasting groups, the mean difference between groups was 0.6 with standard deviation = 0.7 and power 80% and type I error type 0.05 (α=0.05) and dropout rate 20%, the sample size was considered to be 26 in each group. However, two participants dropped out of the fasting group, thus we chose fasting subjected selection (n=24) and non-fasting selection (n=26). MedCalc Statistical Software version 18.2.1 (MedCalc Software bvba, Ostend, Belgium) was used to compute sample size. Quantitative continuous variables were described using mean± standard deviation (SD). Considering clinical interpretation and dynamic and covary changes of biomarkers, Factor analysis through principal component analysis with varimax rotation was employed for a combination of measured biomarkers. The Mann-Whitney test was used to compare quantitative variables between the groups. Statistical significance was considered at the level of 0.05 for all tests. All statistical analyses were conducted by using SPSS statistical software (version 22, SPSS Inc. Chicago, IL).

## RESULTS

The demographic of the 50 participants of the study is presented in the supplementary file (Table S2). The majority (88%) were married and about 58% of participants were <45 years old. There were equal numbers of males and females, 62% of them with university degrees and 38% with technical diplomas degrees. Many of them (64%) had academic jobs while only 36% had non-academic employment. The proportion of normal-weight and overweight participants was 92, and 8%, respectively. As depicted in Table S2 there is no significant differences between fasting and non-fasting groups in the level of physical activities during the holly Ramadan. 

**Figure 2:**
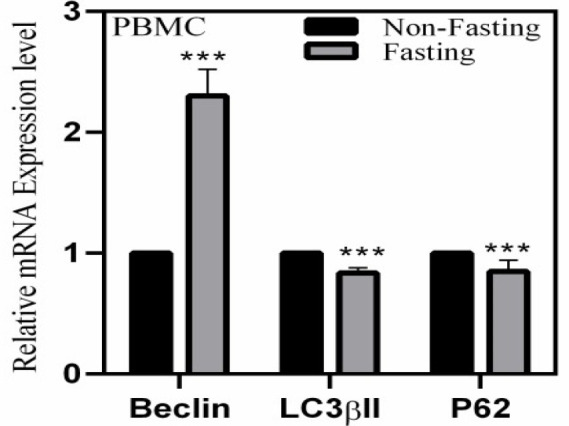
The relative mRNA expression levels of *Beclin-1*, *LC3 *and *P62* in fasting and non-fasting groups. The Data are reported as the means ± SD of three independent assays (n=3, ***P<0.001)

The results of the effect of Ramadan fasting on inflammatory (IL-6 and hsCRP) and autophagy (Beclin-1 and LC3) markers are presented in Table 1. There was a significant increase in the serum levels of IL-6 or hsCRP in non-fasting as compared to fasting groups for male and female participants (*p*>0.05). There was no significant difference in the fasting group’s Beclin-1 and LC3 serum levels compared to the non-fasting (*p*>0.05). Table 1 indicated a non-significant upward trend for Beclin-1 and LC3 among the total fasting group compared to the non-fasting group. While based on the result fasting women had higher Beclin-1 in serum (746.6±135.8) compared with non-fasting women (573.4±249) (*p*=0.031).  To investigate the impact of fasting on the gene expression of autophagy markers, the mRNA levels of Beclin-1, P62, and LC3 were tested by RT-PCR. As illustrated in Figure 2, Beclin-1 mRNA expression increased significantly (2.30-fold, P<0.001), while expressions of LC3II and P62, markers of autophagy flux, showed notable decreases (approximately 16%, *p*<0.001, and 15%, *p*<0.001, respectively) when comparing the total fasting group to the non-fasting group.

The mean and standard deviation of age in fasting and non-fasting groups were 42.8±9.1 and 41.3±12.48years, respectively. The changes in biochemical factors are described in the supplementary file (Table S3), which shows that there were no significant differences in biochemical variables between fasting and non-fasting groups (*p*>0.05) except for BUN level. Interestingly, a higher BUN level was determined in the fasting group compared with the non-fasting one (13.91±3.62 vs. 11.73±3.86 mg/dl, respectively, *p*=0.045). According to statistical analysis, no difference was detected even among men or women in our study groups. In this study, we analyzed routine hematological factors including Hb, RBC, WBC, and PLT counts in whole blood of fasting and non-fasting groups. As shown in the supplementary file (Table S4), no significant changes were detected in hematological parameters after one month of Ramadan fasting (*p*>0.05).

## DISCUSSION

Growing data suggests fasting can alter cellular metabolism from glucose to ketone and increase health, longevity, and stress resistance [9]. Many studies have examined the health effects of Ramadan fasting, however the results are occasionally conflicting. The effects of Ramadan intermittent fasting on Alzheimer's, malignancies, metabolic syndrome, inflammation, and neuropsychiatric illnesses have been supported by various studies [33]. Several original investigations, systematic reviews, and meta-analyses have examined healthy Ramadan fasting [1, 5, 34, 35]. The evidence suggests that Ramadan fasting affects several key health markers. The effects on glucose homeostasis have been studied, with mixed findings [27]. Ramadan fasting also affects body weight [27, 36], body composition [7], and fat mass—particularly visceral fat [37]. During Ramadan, inflammatory and oxidative stress indicators, immunomodulatory reactions, and serum lipid levels change [38, 39]. We found no biological differences between healthy fasting and non-fasting volunteers. 

A report found that fasting during Ramadan does not affect biomedical variables including uric acid, serum electrolytes/lipid/glucose concentration, cellular damage biomarkers, immunelogical, or inflammatory biomarkers in healthy bodybuilders [40]. Another study found that fasting and physically active males had metabolic changes during Ramadan, but not non-fasting groups. This change added to urine specific gravity, urea, uric acid, salt, chloride, and HDL cholesterol [41]. Beltaief study found that biochemical indicators like TG, blood glucose, and cholesterol improved post-Ramadan in 517 high-risk cardiovascular disease patients [42]. A Dubai study on healthy Muslims from three nationalities found no significant change in fasting TC, TG, HDL, and LDL in Sudanese and Emirati groups by Ramadan. Pakistani women had a 6% increase in TC [43]. 

These data suggest that fasting individuals' metabolic parameters do not change after Ramadan. The latest study includes a 30-day Ramadan fast and proteomic analysis of serum samples from healthy volunteers. The findings demonstrated significant overexpression of regulatory proteins involved in immune system function, cytoskeleton remodeling, DNA repair, lipid and glucose metabolism, circadian clock regulation, and cognitive function. This vast range of elevated proteins suggests that protracted fasting benefits several biological pathways [44]. A systematic review and meta-analysis by M. A. Faris et al. examined how Ramadan diurnal intermittent fasting (RDIF) affected glucometabolic markers in healthy, non-athletic Muslims. A search of several databases yielded 72 research from 1982 to 2020 with 3134 participants from 22 countries. Fasting glucose (FG), insulin, HOMA-IR, leptin, and adiponectin were measured. RDIF-induced glucometabolic indicator changes were usually advantageous, according to the meta-analysis. During Ramadan, fasting glucose (FG) fell considerably, suggesting glucose management improved. RDIF exhibited no statistically significant effects on serum insulin, HOMA-IR, leptin, or adiponectin. In particular, serum insulin and adiponectin levels rose slightly [45].

Fasting groups had significantly higher BUN levels as nitrogen end products of metabolism than non-fasting groups. Food restriction may boost protein catabolism to provide a glucose basis during 12-14 hours of fasting to prevent hypoglycemia. After Ramadan, diabetics' BUN levels rose [46]. Our data showed no significant changes in serum BUN/Creatinine ratio, a renal function indicator, between groups. Ramadan fasting did not harm renal function in stage III or IV CKD and hemodialysis patients [47, 48]. Several studies show that fasting upregulates critical regulatory proteins and signaling pathways in lipid and glucose metabolism, DNA repair, circadian clock, immune system, cytoskeleton remodeling, and cognitive function [49, 50]. Food restriction can also upregulate autophagy, a crucial cellular system that degrades unneeded and damaged cytoplasmic components. Autophagy, a "double-edged sword" signaling system, has garnered interest recently. To compensate for stress, lysosomes assist cells maintain hemostasis [51]. If cells cannot handle stimuli, autophagy can activate an apoptotic signaling pathway. Periodic fasting stimulates cell autophagy and activates progenitor cells, rejuvenating cells [52].

Recently, autophagy has been shown to defend against cancer, diabetes, neurodegeneration, heart, and aging [51, 53]. Lacking autophagy genes (Beclin-1, Atg 7) can cause metabolic abnormalities as glucose intolerance, fat buildup, muscle atrophy, and early mortality [54]. Calorie restriction has been shown to enhance life span through autophagy in several organisms

Research reveals that several diet models reduce inflammation, burn fat, and boost the immune system, prolonging animal and human lifespans [55]. Starvation activates autophagy in Ramadan fasting to eliminate unfolded and misfolded proteins, aged and defective organelles, and cell membranes to restore health. Fasting during the holy month enhanced Beclin-1 expression, according to our findings. Reduced Beclin-1 expression and activity are linked to cancer, neurological disorders, cardiomyopathy, and aging [56]. Ramadan fasting is intermittent because water and food are not eaten from dawn to sundown. Due of food restriction, it strongly stimulates autophagy flux [57]. Fasting-mediated autophagy has a valuable role in restoring the hemostasis of multiple tissues and organs [51]. 

A recent study found that intermittent fasting can induce autophagy and exert neuroprotective effects after spinal cord injury. Ramadan's effects on autophagy have not been highlighted. As per our findings, fasting promotes autophagy by increasing the expression of LC3βII, Beclin-1, TFEB, and AMPK/mTOR pathways [58]. In 2015, Rebecca Godar claimed that intermittent fasting in adult C57BL/6 mice triggered autophagy by nuclear translocation of TFEB, which started the autophagy-lysosome machinery and protected cardiac ischemia-reperfusion injury [59]. A time-restricted feeding (TRF) study found that LC3A, an autophagy structural component, rose after an 18-hour fast before breakfast [60]. They claimed that TRF may increase insulin secretion, glucose levels, and anti-aging through circadian and fasting-related processes, including the autophagy pathway. The mRNA expression levels of Beclin-1, LC3, and P62 in PBMC after one month of fasting were examined to assess autophagy in our study. The fasting group had higher Beclin-1 expression and lower LC3 and P62 expression. Stress activates autophagy, according to these studies. 

We found that Ramadan fasting, like other therapeutic fasting programs, induces autophagy (Beclin-1 expression and P62 degradation) as a stress-reduction signaling mechanism. Additionally, the fasting group had a non-significant increase in Beclin-1 and LC3 serum levels compared to the non-fasting group. Our serum and gene expression data of autophagy markers suggest Ramadan fasting has modified autophagy. Because Beclin-1 increases, Ramadan fasting may be a stressor that activates autophagy in active mode to compensate for the loss of important cellular metabolites and energy. Our serum autophagy marker levels did not vary as much as gene expression in experimental groups. We found a significant increase in Beclin-1 gene expression and a decrease in LC3 and P62 (an autophagy activation indicator) in PBMC, but only a trend in serum. To enhance the protein level of any component, we need longer time to make and release it in the blood. The presence of serum proteases and the short half-life of protein lead serum and cell factor quantities to differ, although this dispute alludes to ELISA's sensitivity limitations. The minimum detectable amount of protein factors in ELISA is greater than gene expression in quantitative real-time PCR. Serum Beclin-1 levels were substantially lower in fasting women (573.4) than fasting men (1798.5). Different estrogen levels in men and women may explain these sex differences in autophagy marker serum levels. It has been established that estrogen levels regulate basal autophagy and mitophagy through numerous signaling pathways. These findings show that estrogen and its receptor-mediated autophagy alter cell fate and human illnesses [61, 62]. Qinghai Menget et al. confirmed that in post-menopausal women, decreased estrogen levels lead to decreased ERα expression, autophagy, and inflammation, causing severe damage to the ascending aorta [63]. It has been mentioned that in several diseases including Alzheimer's [64], cancer [65], osteoporosis [66], and so on [67, 68] the differences in the axis of E2–ERα–autophagy contribute to gender differences. Estradiol signaling pathways interact with visceral fat mass and ERα expression, making females more susceptible to illnesses through autophagy. 

The present study was one of the first to completely assess Ramadan fasting's effects on multiple indicators, however it had limitations. Ramadan fasting affects health in many ways, including nutrition and lifestyle changes. Classically comparing all measures before and after Ramadan in people under research regarding natural changes in basal metabolism is best. Comparing each person to himself/herself would have helped us understand and conclude our results, especially autophagy markers. Our study used a controlled cohort strategy to collect data once. Future research should explore controlling the individual's food, physical performance, and sleeping habit during this month for the best outcomes. Since these pathways govern autophagy under fasting, Ramadan fasting should be studied on oxidative stress, endoplasmic stress, and UPR. 

We performed the first study to address how Ramadan fasting affects the autophagy process in healthy humans. Based on these findings Ramadan fasting does not have any adverse effects on biochemical, hematological, and inflammatory parameters in healthy people. Considering our data, we proposed that one of the cellular contributing factors that is affected by Ramadan fasting might be the modulation of the autophagy process. Initiation of autophagy can compensate reduction in energy and vital metabolites in the face of food restriction during Ramadan to restore cellular homeostasis and consequently improve health. However, definitive evidence for this claim requires further research, including analysis of ATG genes and measurement of autophagy marker proteins using immunoblotting assays. Future studies are expected to address these aspects.

**Table 1:** Inflammatory and autophagy markers in non-fasting (control) and fasting groups.

**Variants**	**Total**			**Female**			**Male**		
	**Fasting** n=24	**Non-fasting** n=26	***p***	**Fasting** n=10	**Non-fasting** n=15	***p***	**Fasting** n=14	**Non-fasting** n=11	***p***
**Beclin-1 (pg/ml)**	1288±1479.7	757.2±124.2	0.69	573.4±249	746.6±135.8	0.03	1798.5±1776.9	770.8±112.6	0.22
**LC3 ** **(pg/ml)**	258.2±182.4	207.7±95.1	0.91	169.2±44.3	196.6±50.2	0.13	321.7±217	221.8±134.3	0.37
**IL6 ** **(pg/ml)**	3.5±2.6	3.8±2.3	0.29	4.1±2.7	4.2±2.3	0.99	3.1±2.5	3.4±2.3	0.22
**hsCRP ** **(µg/ml)**	0.8±0.5	1.1±1.4	0.40	1±0.7	1.2±1.6	0.39	0.6±0.3	0.9±1.1	0.781

Data presented as mean ± standard deviation; pg/dl: Pico gram per Deciliter; µg/dl: Microgram per Deciliter.
