# Supplementary material for: Effect of 30-day Ramadan fasting on autophagy pathway and metabolic health outcome in healthy individuals
Source: Mol Biol Res Commun. 2025;14(2):115–27. doi: 10.22099/mbrc.2024.50105.1978 (PMC11865935; doi:10.22099/mbrc.2024.50105.1978)
Supplement: Supplementary file 1 [file MBRC-14-115-s001.pdf]

Table S1: The Primer sequences of autophagy markers

Genes	Forward primers (5'-3')	Reverse primers (5'-3')
LC3	AACGGGCTGTGTGAGAAAAC	AGTGAGGACTTTGGGTGTGG
P62	AATCAGCTTCTGGTCCATCG	TTCTTTTCCCTCCGTGCTC
Beclin-1	AGCTGCCGGTTATACTGTTCTG	ACTGCCTCCTGTGTCTTCAATCTT
GAPDH	CGACCACTTTGTCAAGCTCA	AGGGGTCTACATGGCAACTG

Table S2: Sociodemographic of the study population (n = 50). p-value of Chi-square test Analysis of mean (w2 test)

Sociodemographic variable	N (%)	non-fasting(%)	Fasting(%)	P-value
**Age (year)**				
**20-٣2**	4 (8)	3 (12)	1 (4)	0.63
**٣3-44**	25 (50)	12 (46)	13 (54)	
**45 and above**	21 (42)	11 (42)	10 (42)	
**Sex**				
**Male**	25 (50)	11(42)	14 (58)	0.25
**Female**	25 (50)	15 (58)	10 (42)	
**Marital status**				
**Single**	6 (12)	4 (15)	2 (8)	0.44
**Married**	44 (88)	22 (85)	22 (92)	
**Education level**				
**Technical diploma**	19 (38)	10 (38)	9 (38)	0.94
**University**	31 (62)	16 (62)	15 (62)	
**Type of Employment**				
**Academic**	32 (64)	15 (58)	17 (71)	0.33
**Non-academic**	18 (36)	11 (42)	7 (19)	
**Weight (kg)**				
**45-55**	8 (12)	5(19)	3 (12)	
**55-65**	16 (32)	8 (27)	8 (38)	0.81
**65-75**	26 (52)	13 (54)	13 (50)	
**Physical Activity**				
**Low**	17 (34)	9 (35)	8 (33)	
**Moderate**	30 (60)	15 (58)	15 (63)	0.88
**High**	3 (6)	2 (7)	1 (4)	
**BMI (kg/m^2)**				
**Normal (18.5-24.9)**	46(92)	23(85)	23(96)	0.33
**Overweight (25.0-29.9)**	4 (8)	3(15)	1(4)	
**Obese (>30)**	-	-	-	

BMI, body mass index (kg/m2).

Table S3: Biochemical parameters in non-fasting and fasting groups.

Variants	Total			Female			Male		
	**Fasting** n=24	**Non-fasting** n=26	***P* Value**	**Fasting** n=10	**Non-fasting** n=15	***P* Value**	**Fasting** n=14	**Non-fasting** n=11	***P* Value**
**FBS (mg/dl)**	108±68.1	105±33.7	0.20	129.8±104.5	110.1±43.2	0.76	92.5±5.4	98.1±11.5	0.16
**HbA1c (mg/dl)**	5.6±1.5	5.6±1.2	0.58	6±2.3	5.8±1.5	0.53	5.3±0.3	5.4±0.6	0.85
**TG (mg/dl)**	128.9±44.2	141.9±75.2	0.97	119.9±38.8	129.2±54.5	0.89	135.4±48	159.3±96.9	0.93
**Chol (mg/dl)**	183.8±38.1	180.4±34.6	0.93	190.8±36.9	179.7±19.8	0.93	178.8±39.6	181.3±49.5	0.97
**Uric (mg/dl)**	5.2±1.5	5.3±1.2	0.54	4.3±1.1	4.6±0.9	0.56	5.8±1.5	6.3±1	0.24
**HDL (mg/dl)**	47±7.9	48.3±8.7	0.47	52.3±8.3	51.8±8.5	0.97	43.3±5.2	43.5±6.4	0.80
**LDL (mg/dl)**	95.5±29.3	95.2±17.1	0.98	101±21.7	93.4±14.2	0.80	91.6±34	97.7±21	0.80
**BUN (mg/dl)**	13.9±3.6	11.7±3.8	0.04	12.6±3.2	11.4±3.9	0.42	14.8±3.7	12.1±3.8	0.10
**Cr (mg/dl)**	1±0.1	1±0.1	0.77	0.9±0.0	0.9±0.2	0.93	1.1±0.0	1.1±0.1	0.08
**Ca (mg/dl)**	9.1±0.5	9±0.5	0.69	9.1±0.4	8.9±0.2	0.26	9.1±0.6	9.2±0.7	0.29

**P (mg/dl)**	3.4±0.5	3.6±0.4	0.17	3.5±0.4	3.6±0.3	0.99	3.3±0.5	3.7±0.5	0.09
**Protein (g/dl)**	7.1±0.5	7.2±0.4	0.63	7.2±0.3	7.2±0.4	0.76	7.1±0.5	7.4±0.3	0.26
**Alb (g/dl)**	4.4±0.3	4.4±0.2	0.73	4.4±0.3	4.3±0.1	0.49	4.4±0.4	4.6±0.2	0.40
**Globulin (g/dl)**	2.7±0.2	2.8±0.4	0.14	2.8±0.2	2.9±0.4	0.60	2.6±0.2	2.7±0.3	0.20
**ALT (IU/L)**	25±17.7	20.7±10	0.52	17.9±9.5	17.5±6.9	0.68	30.1±20.6	25±12.1	0.93
**AST (IU/L)**	18.4±8.3	18.8±10.9	0.75	13.2±5.3	15.8±5.7	0.31	22±8.1	22.8±14.9	0.43
**ALP (IU/L)**	210.2±79.1	207.7±63.1	0.96	218.9±63.8	214.6±71.4	0.68	204±90.3	198.3±51.5	0.85
**Total-Billi (mg/dl)**	0.8±0.3	0.7±0.2	0.33	0.7±0.1	0.7±0.2	0.89	0.9±0.3	0.8±0.1	0.31
**Direct-Billi (mg/dl)**	0.4±0.2	0.4±0.1	0.54	0.3±0.1	0.3±0.1	0.68	0.5±0.2	0.4±0.1	0.60
**Indirect-Billi (mg/dl)**	0.4±0.2	0.4±0.2	0.49	0.4±0.1	0.4±0.2	0.49	0.4±0.2	0.4±0.2	0.76

Data presented as mean ± standard deviation; mg/dl: Milligram per Deciliter.

Table S4: Hematological parameters in non-fasting (control) and fasting groups.

Variants	Total			Female			Male		
	Fasting n=24	Non-fasting n=26	*P* Value	Fasting n=10	Non-fasting n=15	*P* Value	Fasting n=14	Non-fasting n=11	*P* Value
**Hb (gm/dl)**	15±1.9	14.6±1.7	0.52	13.5±1.4	13.4±1	0.89	16.2±1.3	16.3±0.8	0.69
**RBC ($x10^6/mm^3$)**	5.4±0.6	5.3±0.5	0.41	5±0.4	5.1±0.4	0.76	5.8±0.5	5.6±0.4	0.15
**WBC ($x10^6/mm^3$)**	6±1.4	6.5±1.7	0.46	6.7±1.6	6.2±1.9	0.33	5.5±1	6.7±1.6	0.11
**PLT ($/mm^3$)**	288681.8±84042.1	260307.7±61239	0.24	350300±82856	259266.6±63226.8	0.00	237333.3±39232	261727.2±61441.1	0.41

Data presented as mean ± standard deviation; mg/dl: Milligram per Deciliter.
